# Atlanta Academy of Medicine

**Published:** 1878-11

**Authors:** 


### ATLANTA ACADEMY OF MEDICINE.

ATLANTA, October 28, 1878.

Dr. J. F. Alexander, President, in the chair.

Dr. George H. Rohe exhibited an apparatus for the inhalation of ether. He remarked that the unpleasant effects of chloroform inhaled to produce anæsthesia, had induced him to seek something safer. With this view, he had concluded to use ether in preference, and in looking around had found what is called a sponge bag. This suggested the propriety of so regulating the inhalation that comparative safety may be secured by making an apparatus which will afford the greatest amount of the anæsthetic in the shortest space of time. This will, of course, afford insensibility with less amount of ether than when taken more slowly. Poisoning, therefore, is less likely to occur. The apparatus presented to the Academy is intended for ether only, and so arranged that no air is inhaled along with the ether, which Dr. Rohe thinks accounts for the readiness of producing anæsthesia so speedily and safely. Complete insensibility may be produced in two or three minutes and with two or three ounces of ether. The inhaler exhibited consists of a cloth bag lined with a thin coat of rubber, and containing a loose piece of flannel for the absorption of the ether when poured into the bag. A mouthpiece is attached to the bag, which is so perfectly fitted to the face that no air can enter along with the inhaled ether. Dr. R. inclines to the rejection of chloroform entirely as an anæsthetic, and does not at all approve of a mixture of the two.

Dr. Baird, while he very highly approved of Dr. Rohe's apparatus, was inclined to object to his sweeping denunciation of chloroform. He thinks that with proper caution chloroform may be used safely, and in certain cases is preferable to ether.

Dr. Rohe, in answer, remarked that there are usually exceptions to most general rules, and in this case there are exceptions, such as the use of actual cautery about the face, making ether objectionable.

Dr. Lowe expressed satisfaction at the exhibition of Dr. Rohe's inhaler, and found many points of interest connected with it.

Dr. Baird reported favorable progress in the treatment of the case of diphtheria, reported at last meeting. He also alluded to the case of so-called idiopathic tetanus. A recent discovery in the case is likely to lead to a favorable termination. On examination it was ascertained that slight adhesion of the prepuce to the corona glandis existed, and the attempt at retracting the foreskin induced a violent paroxysm. The second attempt had the same effect, and not until the patient was completely anæsthatized could the examination proceed and the adhesion be broken up. Since this relief from the unnatural condition of the prepuce the patient gives encouraging evidences of improvement.

---

Atlanta, November 4, 1878.

Dr. Calhoun presented a specimen of an injured eye, by a shot entering the nasal side of ball just in rear of the cornea. Enucleation of the ball was determined upon, on account of evidences of sympathetic inflammation having made their appearance. Dr. C. called attention to the fact that such injuries almost invariably cause blindness, not only of the injured, but of the uninjured eye. In answer to the question whether the removal of an injured eye is a certain protection to the other, said that it is generally certain protection to the sound eye, if not delayed too long. Cases were mentioned, in which sympathetic inflammation of the sound eye commenced at various periods after the injury, from a few days to thirty or fifty years. He also reported a case of diphtheria of the conjunctiva, which he treated by the local application of sulphur and internal use of iron. The treatment was commenced yesterday, and to-day decided improvement is apparent.

Dr. Rohe inquired whether there were evidences of connection between the injury in one eye with the loss of sight and in the other, after the lapse of fifty years, and whether other causes may lead to the second difficulty? To which Dr. Calhoun replied that such may sometimes be the case, but from certain symptoms the sympathetic origin may be determined.

Dr. Baird reported progress in the treatment of the so-called idiopathetic tetanus. After the last report the prepuce was slitted, and improvement seemed to progress satisfactorily; afterwards for two days was not doing so well as before. Since that time improvement is marked, and the prospect of recovery is now flattering.

Dr. Grant presented specimens of medicinal indigenous plants, and gave some of the therapeutic results to be expected from their use. The leaves, stems and flowers of the plants are arranged carefully on paper boards, so as to preserve their identity as clearly as possible. Some three hundred medicinal plants have been collected from different points in Georgia, mostly in the neighborhood of Atlanta, and preserved in this way by Dr. Grant.

